# Big Data and the Little Big Bang: An Epistemological (R)evolution

**DOI:** 10.3389/fdata.2020.00031

**Published:** 2020-09-18

**Authors:** Dominik Balazka, Dario Rodighiero

**Affiliations:** ^1^Center for Information and Communication Technology (FBK-ICT) and Center for Religious Studies (FBK-ISR), Fondazione Bruno Kessler, Trento, Italy; ^2^Comparative Media Studies/Writing, Massachusetts Institute of Technology, Cambridge, MA, United States; ^3^Berkman Klein Center for Internet & Society, Harvard University, Cambridge, MA, United States

**Keywords:** big data, power dynamics, knowledge discovery, epistemology, sociology

## Abstract

Starting from an analysis of frequently employed definitions of big data, it will be argued that, to overcome the intrinsic weaknesses of big data, it is more appropriate to define the object in relational terms. The excessive emphasis on volume and technological aspects of big data, derived from their current definitions, combined with neglected epistemological issues gave birth to an objectivistic rhetoric surrounding big data as implicitly neutral, omni-comprehensive, and theory-free. This rhetoric contradicts the empirical reality that embraces big data: (1) data collection is not neutral nor objective; (2) exhaustivity is a mathematical limit; and (3) interpretation and knowledge production remain both theoretically informed and subjective. Addressing these issues, big data will be interpreted as a methodological revolution carried over by evolutionary processes in technology and epistemology. By distinguishing between forms of nominal and actual access, we claim that big data promoted a new digital divide changing stakeholders, gatekeepers, and the basic rules of knowledge discovery by radically shaping the power dynamics involved in the processes of production and analysis of data.

## Introduction

The former director of the *Oxford Internet Institute*, Luciano Floridi, claims that while 180 exabytes of data were collected between the *invention of writing* and 2006, in 2011, they grew up to 1,600 exabytes (Floridi, 2012, p. 435). Two years later, Andrej Zwitter argues that while 5 billion gigabytes were collected between the *beginning of recorded history* and 2003, the same amount was generated every 2 days in 2011, estimating 5 billion gigabytes every 10 s in 2015^1^ (Zwitter, 2014, p. 2). Despite the different approximations between Floridi and Zwitter, data collection is constantly and exponentially growing “at a rate between 40 and 60% a year” (Bughin, 2016, p. 1).

This unprecedented abundance has been addressed over the years using expressions such as *deluge* (Anderson, 2008; Bell et al., 2009) or *avalanche* (Miller, 2010). The experts declare that big data are provoking a *computational turn* (Lazer et al., 2009; Berry, 2011), leading toward a *fourth paradigm* of science (Kelling et al., 2009; Chandler, 2015), a sort of *quiet revolution* (Bollier, 2010) capable of transforming how we live, work, and think (Mayer-Schönberger and Cukier, 2013a), opening the door to the Petabyte Age (Anderson, 2008; Manovich, 2011).

First references to “big data” appear already in 1993 (see Table 1), but it is only in 2012 that the literature about the topic started to grow exponentially. Despite the increased relevance of the subject and the various challenges raised by big data, papers that engaged directly and explicitly with underlying epistemological issues remain a minority—roughly 0.5% of publications.

**Table 1:** Number of papers about “big data” by year and references to epistemology as of 24 June 2020, 1980–2020.

	**Refers to epistemology?**		
**Year**	**No**	**Yes**	**Total**
1993	1	0	1
1994	1	0	1
1995	0	0	0
1996	1	0	1
1997	0	0	0
1998	1	0	1
1999	3	0	3
2000	1	0	1
2001	4	0	4
2002	1	0	1
2003	3	0	3
2004	6	0	6
2005	2	0	2
2006	7	0	7
2007	4	0	4
2008	16	0	16
2009	16	0	16
2010	17	0	17
2011	31	0	31
2012	284	2	286
2013	1,325	2	1,327
2014	2,904	4	2,908
2015	5,620	19	5,639
2016	7,511	30	7,541
2017	8,561	38	8,596
2018	9,536	41	9,577
2019	9,154	38	9,192
2020	3,503	16	3,519

*Results based on Web of Science: Science Citation Index Expanded; Social Sciences Citation Index; Arts and Humanities Citation Index; Conference Proceedings Citation Index—Science; Conference Proceedings Citation Index—Social Science and Humanities; Emerging Sources Citation Index. The query considers title, abstract, author keywords, and keywords plus*.

We are not suggesting that lack of epistemological debate implies lack of methodological concerns. There are numerous papers that discuss big data-related issues without connecting them to methods, scope, or validity of a presumably new paradigm in the theory of knowledge. However, this is precisely the heart of the matter. A new paradigm was frequently invoked, occasionally outlined, but it needs further developments. Researchers self-assessed a radically new and independent status of the big data field, claiming a considerable autonomy for themselves, but without managing to justify this conceptual move and without establishing new epistemological standards.

## What Qualifies as Big Data?

The scientific community is struggling to reach a shared definition that currently does not exist. On the other side, popular and widespread sources, like the Oxford English dictionary or Wikipedia, use the term *big data* when the traditional modes of computational storage and analysis are not sufficient to deal with large datasets. In other words, big data are big. The concept of volume is widely employed in scientific literature as well, and it occasionally becomes the sole defining feature (Manovich, 2011; Strom, 2012; Jenkins, 2013; Taylor et al., 2014). However, the use of the term *volume* implies two major problems. First, the epistemological problem is identified through technical issues such as storage and maintenance (Strom, 2012; Trifunovic et al., 2015), underestimating the bias that collecting and processing data imply. In this perspective, which promotes a structured epistemological myopia, increasing the computational power is all we need to solve, once and for all, the challenges raised by big data (see Mercer, 2019). However, epistemological issues require epistemological solutions (Floridi, 2014). Second, the volume of big data is still widely undefined. Kitchin and McArdle (2016) observe that defining this threshold is not easy. Moreover, the volume of a dataset can be measured using the number or the size of records producing different results.

The inconsistency of these definitions makes the entire phenomenon blurry, providing a safe ground to affirm that big data were employed for centuries (Arbesman, 2013; Kaplan and di Lenardo, 2017). While the volume is not relevant as much as the velocity and the exhaustivity that *usually* characterize big data (*ivi*, Kitchin and McArdle, 2016), the discussion about volume is, in reality, a discussion about perception. The point is not how we measure but rather how we perceive a dataset. Data abundance indeed is perceived through the “technologies [that] were invented to deal with the perceived overload” (Strasser, 2012, p. 85). Being big thus becomes a *historically contextualized* quality that a dataset might have with regard to the technologies available in a specific time period (Lagoze, 2014). Although the current amount of available information was never experienced before, this was equally veritable in many moments of human history. It is sufficient to think, for example, about the specimen of 17,000 argyle tablets recording administrative data that were produced in the ancient city of Ebla between II and III millennium BC (Kaplan and di Lenardo, 2017), and consider the massive impact that movable type had on the velocity of the printing process and on the volume of printed material during the so-called “printing revolution” of 1,455 (Eisenstein, 1983). So, what makes the current overload so different from the previous ones?

Concepts such as velocity, variety, and veracity provide a less tautological definition (Laney, 2001; Floridi, 2012; Arbesman, 2013; Lowrie, 2017). Big data are so defined as large datasets generated in real time, characterized by messiness and by different types of content such as images, text, or numbers. “Versatility, volatility, virtuosity, vitality, visionary, vigor, viability, vibrancy, and even virility” are other concepts employed by scholars (see Uprichard, 2013, p. 1). The variety of nuances supposed to have indexical power, as noted by Emma Uprichard, makes the substantial lack of agreement in the scientific community clear. This thesis is also supported by Kitchin and McArdle (2016), who compared 26 datasets labeled as “big data” according to volume, velocity, variety, exhaustivity, resolution and indexicality, relationality, extensionality, and scalability. None of these traits was present in all datasets. Since big data do not share common traits, only *prevailing* ones, Kitchin and McArdle argued that big data do not constitute a genus but belong to different species (*ivi*, Kitchin and McArdle, 2016), yet how can these species be defined if their common genus cannot be isolated? It is dangerous to define and classify species in the absence of any unifying characteristic.

An alternative set of approaches adopted a slightly different perspective. Mayer-Schönberger and Cukier, for example, stress how big data create a shift from a causal approach to knowledge discovery, to an approach based on inductive reasoning and correlation (Mayer-Schönberger and Cukier, 2013a). Similarly, Boyd and Crawford claim that big data are not just a technological issue but also a cultural and scholarly phenomenon (Boyd and Crawford, 2012, p. 663). These definitions suggest that big data should be classified according to the way they are used and perceived, rather than their intrinsic characteristics. If presumably defining features, like volume or velocity, lack indexical power and are historically contextualized, then a relational approach might represent an important step toward a shared definition capable of distinguishing big data from lots of data.

The epistemological problem is concerned with the way big data are used to produce and justify knowledge. To approach the puzzle, it is thus important to examine the complex relations between produced knowledge, knowledge producers, and means of knowledge production. What exactly constitutes such means in big data research, however, is currently unclear. Since the meaning of big data still works as an umbrella for a multitude of different theoretical solutions (Favaretto et al., 2020), the problem of definition remains inherently bound to the epistemological one. Lots of data are mixed up with big data, evolutionary and revolutionary aspects are blended together, and a strong objectivistic rhetoric is minimizing the challenges raised by the scientific discussion.

## The Promise of Revolution: Positivism in Incognito

At a deeper level, technocentric definitions that ignore epistemological issues have led to a diffused overconfidence in the exactitude of data. Today, big data form an emerging field pervaded by the mantra “let the data speak.” Many practitioners invoke a *paradigm shift*, oriented toward an utterly new epistemological and methodological answer based on Kuhn's concept of scientific revolution (Kuhn, 1962). Using a provocative terminology, Chris Anderson announced the Petabyte Age in which figures “speak for themselves” without any previous knowledge involved. Asking what scientists can learn from Google, Anderson opens the door to a data-driven and -intensive approach to intelligent computation (Anderson, 2008).

During the following years, big data have been employed by universities and companies to identify universal laws (Lehrer, 2010; West, 2017) and forecast future trends (Ginsberg et al., 2009), ignoring errors and producing biased results (for an overview, see Lazer et al., 2014; McFarland and McFarland, 2015; Boulamwini and Gebru, 2018; Zunino, 2019).

Five years after the publication of Anderson's article, Viktor Mayer-Schönberger and Kenneth Cukier argued that big data are producing a three-fold revolution: (1) the shift from data-poor to data-rich science makes sampling procedures useless and obsolete; (2) the shift from sampling to *n* = all datasets makes methodological concerns about the *exactitude* of data pointless; and (3) the shift from the *age-old search for causality* to correlation produces a radical change in our understanding of the explanatory process (Mayer-Schönberger and Cukier, 2013a). On the same year, Anderson's former colleague, Ian Steadman, took a step further. Steadman claims not only that “algorithms find the patterns and the hypothesis follows from the data” but also that “we're reaching a point where everyone can use big data” and no expertise is required to be a scientist anymore (Steadman, 2013).

More than a century before, Max Weber identified a triple raid of subjectivity into science: (1) a scientist's personal interests and values guide toward a specific understanding of objects (Weber, 1922, p. 10–16); (2) knowledge has to be intended as a “knowledge from particular points of view” (Weber, 1922, p. 47–49); and (3) the “criteria by which this segment is selected” are inseparable from the cultural framework through which the ultimate meaning is acquired (Weber, 1922, p. 51–52). In Weber's text, the scientific objectivity ceased to be assumed *a priori*, becoming a problematic question firmly connected with the notion of methodological strictness. More than a century later, it seems that big data have definitely solved the issues raised by Weber.

### The Pre-social Output of a Socially Created Process

One of the assumptions that allows for the objectivistic rhetoric of big data is the pre-social origin of collected data. Some authors defend this position believing that data are digital raw *traces* left behind daily deeds and that the problem of subjectivity lies in their analysis and interpretation (Chandler, 2015; Goldberg, 2015; Severo and Romele, 2015; Shaw, 2015; Venturini et al., 2017; Kim and Chung, 2018; Jan et al., 2019; Osman, 2019; Shu, 2020). Other authors rather argue for a pure data-driven approach in which intrusions of subjectivity are entirely ruled out (Kelling et al., 2009; Mayer-Schönberger and Cukier, 2013a). For the latter group, the hypotheses emerge from data excluding any need to know the question in advance. As Johnson (2014) writes, “the constructed nature of data makes it quite possible for injustices to be embedded in data itself,” that is, specific groups are more likely to be represented, or values are embedded in data through design decisions and not all the available information is transformed into data. While Johnson is aware of errors and biases in data collection, he agrees with his colleagues by saying that big data are the solution to a problem circumscribed exclusively to theoretically informed and sample-based datasets.

The first objection to this standpoint rests on the fact that datafication necessarily involves the transformation of a flow into discrete categories. In this process, data are first decontextualized and successively recontextualized to be employed in scientific research. What becomes data is thus only the part of the flow that lends itself to be easily adapted to the process of datafication (Berry, 2011; Leonelli, 2014; Wagner-Pacifici et al., 2015). A second objection is that big data collection remains theoretically informed. Since collections cannot be utterly exhaustive, what to collect and how to collect are design-specific decisions that are embedded in data (Bollier, 2010; Crawford, 2013; Bowker, 2014; Frické, 2014; Kitchin, 2014; Diesner, 2015; Seaver, 2017). Third, those acting as data intermediaries hold the ultimate power in deciding which information will become available, for how long, when, and to whom (Schwartz and Cook, 2002; Zwitter, 2014; Schrock and Shaffer, 2017).

These three objections underline human intervention during data collection and storage. The previously discussed idea of rawness thus rests on two implicit assumptions: that digital traces capture natural actors enacting natural behaviors and that data-collecting algorithms are intrinsically neutral. The first assumption incurs in the signaling problem, that is, the lack of correspondence between social and digital world, and will be discussed in major detail in the following section. The latter assumption is relatively well-known in science and technology studies (see Mowshowitz, 1984); can algorithms really be neutral and objective quantifiers of the social world? Can the problem of subjectivity in data collection be solved? Technology itself does not have preferences nor ideas, but the designer does and influences the way the technology works whether intentionally or not. The faith in objective quantification, or *dataism* (van Dijck, 2014, p. 198), is the belief in the efficiency of a “pseudo omniscient algorithmic deity” (Gransche, 2016, p. 60). Algorithms are not only designed by humans for other humans but also embedded within a capitalist mode of production (Mager, 2011, 2014; Biblić, 2016; Burrell, 2016; Ames, 2018; Caplan and Boyd, 2018; Grosman and Reigeluth, 2019). Google, for instance, remains a “profit-oriented, advertising-financed moneymaking machine” that promotes a “stratified attention economy” and delivers “a distorted picture of reality” (Fuchs, 2011). The same goes for alternative search engines, such as Bing or Baidu, and for other companies, such as Twitter or Facebook (see Gaubert, 2017). In this perspective, data collecting algorithms are constantly changing, theory-laden, and naturally selective human artifacts produced within a business environment.

To maintain problematic assumptions about implicit neutrality is particularly dangerous because it leads to overconfidence in exactitude, underestimation of risks, and minimization of epistemological issues. The situation is made even worse by the fact that algorithms are not stable over time and that their changes remain widely unknown. This undermines our ability to identify instances of misuse of data and threatens two of the basic assumptions of science: comparability and replicability of findings (Gelman, 2013; Lazer et al., 2014; Biblić, 2016; Leonelli, 2018). Moreover, digital memory is *forgetful*. Links easily decay, updates occasionally make older files unreadable, and pages are constantly updated and rewritten (see Floridi, 2014). Once these issues are combined with the volatility of algorithms, it becomes evident that big data blend together three different kinds of potential biases: (1) a rewritten algorithm may be applied in the same context, treating data differently at time points A and B; (2) the same algorithm can be applied in another context, treating data at different time points in the same way, but without considering the influence that the changed online environment exercises on monitored users; and (3) a rewritten algorithm may be applied in a mutated context, mixing together the two problems described above.

By highlighting these issues in big data usage, we are not suggesting that “small data” are unproblematic or less problematic when it comes to comparability or replicability. Comparability is a persistent problem whenever different studies and/or different waves of the same research are involved. Replicability is no different. A study about replicability in economics conducted on 60 papers coming from 13 different journals shows that only 43% of results were replicable (Chang and Li, 2015). A psychology report published by the Open Science Collaboration (2015) likewise shows that only 47% of the considered studies are fully replicable, while an additional 21% produce a “weaker evidence for the original findings despite using materials provided by the original authors.”

It is relatively common to define different standards for scientific research and business. The widespread adoption of online surveys in the private sector, despite severe coverage bias and self-selection issues holding back academic circles, is an example of this attitude. As big data are progressively leaving the private companies which collected them for business purposes—be it through web scraping (ten Bosch et al., 2018), trading platforms (Yu and Zhao, 2019), direct data collection (Poppinga et al., 2012), or publicly available sources (Chun-Ting Ho, 2020)—they are increasingly used for scientific research and to inform public policy (Ulbricht, 2020). From this perspective, business standards are simply no longer enough to define acceptable data practices.

In conclusion, the expression “raw data” is nothing else but an oxymoron (Bowker, 2014). The rawness of data is made impossible by the selectivity of theoretically informed algorithms, by the instability of the digital memory, by management decisions of data intermediaries, and by the implicit problems of quantification whenever a flow is reduced into a limited set of discrete categories.

### A Photo Stole My Soul: The End of Theory and Other Selected Tales

The second pillar of the objectivistic rhetoric, partially grounded on the previous one, is the idea that big data are exhaustive. Researchers today have more data, a fact that is clear and not harmful by itself. What is problematic is the assumption that *more* means *all*, that is *n* = all. The idea that these datasets do not constitute a subset but are rather an exhaustive representation of social reality leads to an overestimated rhetoric of exactitude:

“*The social science disciplines largely relied on sampling studies and questionnaires. But when the data is collected passively while people do what they normally do anyway, the old biases associated with sampling and questionnaires disappear”* (Mayer-Schönberger and Cukier, 2013a).

Big data are thus not just a selection of raw traces but are rather the collection of all of them (Ekstrom, 2013; Kitchin, 2013, 2014; Walker, 2015; Cheung et al., 2019; Tani, 2019; Taylor and Meissner, 2019; Tian, 2020). Assuming that data are neutral and fully exhaustive, the problem in handling them becomes technical. In this perspective, new technologies, methods, and procedures are all that is needed to cope with big data (see Strom, 2012; Taylor et al., 2014; Trifunovic et al., 2015; Smith, 2019). On the contrary, once we recognize that data are socially created artifacts, the technological and the technical improvements are no longer enough on their own without a careful methodological and epistemological reflection. The position openly in disagreement with the *n* = all assumption can be summarized in four points:

- Even if *n* = all is accepted as correct in a restricted sense (i.e., there is effective access to all data generated by every user on a given platform), big data suffer from a *signal problem* causing a lack of correspondence between the social and the digital worlds (Manovich, 2011; Crawford, 2013; Lewis, 2015; Gransche, 2016);
- Since big data are constantly growing second by second, it is implicitly impossible to examine them in their totality since every time a new analysis is performed new data are, at the same time, generated (Symons and Alvarado, 2016);
- Since specific portions of the population are more or less likely to actively participate in certain online environments, big data are often a biased sample of the population rather than the population itself (Lewis, 2015; McFarland and McFarland, 2015; Chun-Ting Ho, 2020); and
- Due to the implicit selectivity in data collection, big data never represent a complete set of information (Lagoze, 2014; Leonelli, 2014).

These positions see the *n* = all assumption as a mathematical limit which can be approached but not reached. The exhaustivity, described as one of the core features of big data (Kitchin and McArdle, 2016), is thus a highly questionable assumption at very best.

Big data can be generated by natural actors, physical phenomena, and artificial actors (Zwitter, 2014). Natural actors are not necessarily individuals, an account can hide a collective (Park and Macy, 2015), and individuals can have multiple accounts. As a result, non-random errors are constantly embedded in data. Last year's Cambridge Analytica scandal and the case of Russian trolls targeting teens with memes over Facebook prove the extension of such an issue and how artificially certain supposedly *natural* actors can behave. As photography might not be a truthful representation of reality, big data might not be utterly exhaustive nor accurate (Bollier, 2010; Arbesman, 2013; Brooks, 2013; Frické, 2014; Welles, 2014; Bail, 2015; Jones, 2019; Corple and Linabary, 2020; Lee and Cook, 2020). Everything is significant and outliers are difficult to identify; as such, artificial actors cannot always be distinguished from natural ones, online and offline behaviors can differ, there may be multiple users behind an account, etc.

From this point of view, theory is the victim of an ongoing process of mystification that pushes forward a mistaken conceptualization of big data as inherently neutral, unproblematic and objective. As Hargittai writes, big data are reproducing social inequalities in digital form (Hargittai, 2008). It is thus of utmost importance to ask: “Which people are excluded [?] Which places are less visible? What happens if you live in the shadow of big data sets?” (Crawford, 2013). By leaving these unspoken issues tacitly crawling around, crucial questions as the ones formulated by Crawford are not just unanswered but even unasked. The theory is more necessary today than it ever was.

### Let's Let the Raw Meat Speak

No one will ever claim that a piece of meat on a pan will cook itself or that it arrived on the pan all by itself, nor will anybody suggest that every piece of meat implicitly leads toward a specific dish just like that, by itself. It is simple; there is a cook who decides which cut of meat to buy, how to cook it, and what should be the final result in terms of composition and esthetics. Furthermore, the cook's actions and decisions are embedded in a rich sociocultural context that profoundly influences them. However, this seems not to be the case of data processing. No one generates big data, no one analyzes them, and no one interprets them. Big data speak and the scientists listen. Being a cook implies an active effort of comprehension, elaboration, and interpretation. Even when there is a recipe to follow, many factors influence the process, from the selection of ingredients to the plating—cooking thus remains a creative act. For some reason, however, big data users refuse to picture themselves as thoughtful professionals interacting with data, promoting instead an image of scientists as neutral listeners of the concert produced by the world in motion (Anderson, 2008; Kelling et al., 2009; Prensky, 2009; Dyche, 2012; Torrecilla and Romo, 2018).

It has been already discussed how big data are far from being pre-social artifacts and how their exactitude and accuracy should be the object of a critical examination rather than an assumed *a priori*. The third pillar of the objectivistic rhetoric, the myth of speaking data, is no different from the previous two in terms of its inner fragility.

Whether a simple metaphor or not, assuming that data-derived knowledge is a-problematic can be highly problematic in itself. Different analytical strategies are always possible, and each of them can potentially lead to a different conclusion. The specific compromise adopted by a researcher is influenced by a variety of factors like time, money, or previous knowledge. Furthermore, specific organizational and professional subcultures influence data collection, structure the analysis, and guide the interpretation. This is true for traditional scientific research and remains true once big data become a part of it (Gould, 1981; Boyd and Crawford, 2012; Jenkins, 2013; Bail, 2015). In this sense, data are like ingredients which do not directly lead to a specific recipe but merely push the cook in a given direction. Even when the ingredients perfectly fit an existing recipe, the ingredients *never* perform the required actions and *never* substitute for the cook as the ultimate meaning producer. A dataset might likewise facilitate or obstruct specific approaches to a given question, but it will not generate meaning instead of the researcher. Only when the existence of a “pseudo omniscient algorithmic deity” is refused will the datafied world and society live as two separate and substantially different entities (see Gransche, 2016). Even if data were metaphorically able to speak, their language would require much more than passive listeners to be understood and correctly interpreted. While the situation of journalists, political professionals, and other data outsiders, who continue to rely on “inflated accounts of the objectivity of analytics” (Baldwin-Philippi, 2020), did not change much over the years, instances and claims of pure objectivity (see Robinson, 2018; Succi and Coveney, 2019) became progressively rarer to find in scientific research. In fact, in recent years, the talk about “data-scientific objectivity” in big data relied on transparency, replicability, and the presumably shareable nature of decision-making (Williamson and Piattoeva, 2019) to translate standardization into a form of quasi-objective construction of knowledge.

### The Moral of the Story

More than a century after Weber's theories, scientists struggle to reaffirm what used to be taken for granted. Big data critics move along three main argumentative lines: (1) data are not neutral representations of society as they are collected through specific *modes of production* (Mager, 2014); (2) data do not represent the totality of the population but are rather a “misrepresentative mixture of subpopulations” captured in their online environment and subject to various types of biases (McFarland and McFarland, 2015); and (3) the meaning does not emerge from the data itself but is rather from an effort of interpretation performed by fallible human beings (Gransche, 2016). Retracing Weber's thoughts, specific interests are at work in data production and what is accessed is a part of reality from a specific, culturally mediated standpoint.

At an analytical level, big data users might be divided into two different currents of thought. On one side, the objectivistic approach is deeply rooted in the private sector with several representatives from the academic circles. Objectivists variously support the pillars described above, developing and reiterating the rhetoric of neutrality. These forms of empiricism, in particular in their most radical instances, were extensively and repeatedly criticized by the scientific community (see Resnyansky, 2019). Evaluativists question the objectivistic claims of neutrality and promote a critical re-examination of big data's multiple facets. While objectivists view big data as a revolution that solves most of the challenges traditionally established in the scientific domain, evaluativists say that big data shape those challenges, solve some of them, and introduce new ones.

With respect to the past, the big data phenomenon represents both a revolution and an evolution. Some basic assumptions in the philosophy of science are becoming increasingly troublesome to uphold. Highly restricted accessibility to data—linked with great ethical dilemmas—and the constant variation of processing algorithms obstruct both comparability and Popper's via *negativa* (Popper, 1935).

## A (R)Evolving Paradigm

From an epistemological standpoint, the lack of agreement over the definition of big data (Favaretto et al., 2020) is particularly cumbersome. If the underlying question is “how to use big data to produce and justify knowledge?”, then it becomes clear that not being able to univocally circumscribe the central phenomenon is a major impediment. Vague and omni-comprehensive definitions promote confusion which, in turn, promotes an objectivistic rhetoric. The resulting *techno-optimism* was extensively criticized throughout the previous pages.

To further address the issue and counter the diffused hype-related discourses (Vydra and Klievink, 2019), it is first necessary to establish and underline the evolutionary characteristics that link big data to previous knowledge. We will argue that challenges raised by big data require an answer that should come from within the current scientific paradigm and that big data differentiate themselves from small data at a relational level, altering the power dynamics involved in knowledge production.

### Size and Its Struggles

At the turn of the twentieth century, big data were welcomed as a game changer, even though not all of the large datasets were actually new (Lagoze, 2014). Where do big data establish evolutionary links with small data, and which aspects of this supposedly new phenomenon truly break up with the past? This is a key question that requires an answer in order to strip big data of their current ambivalence and ambiguity.

Technological advancement and rapidly increasing connectivity produced a progressively growing amount of data. The sheer quantity of available information is offering great opportunities to science. For example, the availability of real-time data makes it possible to run a timely analysis capable of answering relevant and pressing questions fastening institutional reactions to emerging social issues. Big data also provide a way to study social groups that were traditionally difficult to reach with survey methods (McFarland and McFarland, 2015). On the downside, however, such growth took a toll on the research process, undermining n-sensitive statistical approaches (Lee and Martin, 2015). The data deluge thus delivered a flood of false positives and called for *big methods* (Williamson, 2014; Ahonen, 2015). Most of the traditional statistical methods were designed to deal with small samples collected through survey methods. As the size and the complexity of a dataset increase, assumptions about data are frequently violated and techniques sensitive to the numerosity of cases produce distorted results. While big data are not replacing small data (see Hekler et al., 2019), the applicability of small methods to big data is highly questionable. What is needed is not just a mere technological improvement but rather a change in the way we look at data in a data-rich context. In this sense and at a methodological level, big data require a huge process of renovation that goes well-beyond a mere evolution of small methods.

### Knowledge Discovery

Big data are said to have triggered a shift from a theory-driven paradigm based on hypotheses, experiments, and simulations to a data-intensive exploratory science which is rather collaborative, networked, and data-driven (Bell et al., 2009; Bollier, 2010; Kitchin, 2014; Chandler, 2015; Trabucchi and Buganza, 2019). While big data impacted certain scientific domains more than others (see Kelling et al., 2009), claims about the rise of an entirely new paradigm in knowledge discovery rest on a misleading interpretation of these two paradigms as completely separated and independent (see also Hekler et al., 2019). In fact, past and contemporary research has “always rested on a combination of hypothesis-driven and data-driven methods” (Strasser, 2012, p. 86) and the current *enchantment* with data-driven methods must face the fact that

“*the studies are irreproducible, the data is irreproducible, the data is unreliable, there is a lack of positive and negative controls, there is the inappropriate use of statistics (often leading to results that the investigator ‘likes’), there is the investigator's ignoring of negative results, there is a pro-positive-result publication bias, and more…”* (Frické, 2014, p. 659).

Data-driven science is too *post-hoc* (Frické, 2014, p. 660) but, rather than seeing two radically opposed paradigms, it is possible to see them as two potentially convergent *cultures of modeling* (Veltri, 2017).

With different degrees of emphasis, it was highlighted that big data were also producing a parallel shift from causal models to correlations (Anderson, 2008; Bollier, 2010; Mayer-Schönberger and Cukier, 2013a). Opponents to this view claimed that correlation is only enough for business purposes and stressed the dangers of the emerging “data fundamentalism” (Crawford, 2013; Bowker, 2014; Gransche, 2016). However, it is once again possible to see these two paradigms as overlapping and convergent (Succi and Coveney, 2019). The theory-driven paradigm frequently relies on correlations, while the data-driven paradigm never truly abandoned causal aspirations (see Canali, 2016). Since causality is difficult to prove, theory-driven approaches often stop at correlations. Big data, on the other hand, make correlation-based explanations both more precise and easier to provide but do not exclude *a priori* integration with causal models (Veltri, 2017; Hassani et al., 2018).

Kuhn defined scientific revolutions as “those non-cumulative developmental episodes in which an older paradigm is replaced in whole or in part by an incompatible new one” (Kuhn, 1962, p. 92). At an epistemological level and within the realm of social sciences, we argue that this is not the case of big data: (1) big data epistemology within the scientific literature is still heavily grounded on basic assumptions of the third paradigm and obey the principles developed by Karl Popper; (2) big data are integrating small data and not replacing them; and (3) theory- and data-driven approaches share commonalities that make them potentially convergent rather than radically divergent.

Big data introduce significant changes at multiple levels of the process of knowledge discovery. While from the methodological point of view, the urge for *big methods* is revolutionary in Lagoze's terms, but not in Kuhn's, the perceived radicalness of epistemological changes rests on an excessively polarized view of theory- and data-driven approaches and of their respective implications.

### The New Digital Divide

The match between correlation and causation hides a performative struggle between companies and universities. In this sense, different perspectives on big data separate experts from scientists, causing science to leak from academia (Savage and Burrows, 2007; Lazer et al., 2009; Boyd and Crawford, 2012; Burrows and Savage, 2014). Experts claim to produce better science than scientists challenging explicitly established standards and practices. However, as Strasser rightly pointed out, “this has contributed to an exaggerated trust in the quality and comparability of the data and to many irreproducible results” (Strasser, 2012, p. 86). The fracture between business and academic circles is further reinforced by the parallel fracture between those who are “big data rich,” typically collective actors of private nature, and those who stay “big data poor” (Gelman, 2013; Andrejevič, 2014; Taylor et al., 2014).

The problem of access conceals two radically different issues, the one of *nominal access* to a dataset, that is the effective possibility to gather data to use, and the one of *actual access*, the possibility not just to obtain such data but also to effectively use them. By distinguishing the two types of access to data, it becomes possible to differentiate the problems derived from restricted accessibility to data from the binding effects of not having the required skills to adequately deal with them. While both of these two forms of access are far from being easily reachable, we interpret actual access as more restrictive because, without nominal access to data, it is impossible to exercise it.

Steadman (2013) argued that we will soon reach a point at which everyone will have the possibility to use big data to produce science. Today it is relatively easy to perform some basic analysis on open source data using free statistical software. In principle, everyone can do it and, at least on paper, it is not difficult to extend this argument from small to big data. Nevertheless, from a practical point of view, things are not that easy. Even if the nominal access to big data is incurring a slow but tortuous democratizing transformation that makes it difficult to forecast future trends, a certain degree of professional skills is and will always be required for the analysis (Manovich, 2011; Boyd and Crawford, 2012; Mayer-Schönberger and Cukier, 2013b; Andrejevič, 2014; Williamson, 2014). Due to the complexity of big data, contrary to what Steadman claimed, it is thus much more likely that big data will require *big skills*. The democratic idea of science crushed against an oligarchy of big data users established by limitations in nominal access and perpetuated by issues of actual access. This characteristic of big data is seriously threatening both the transparency and the replicability of scientific procedures by marking the mismatch between research ethics and *big methods* (Lewis, 2015; Levy and Johns, 2016; Metcalf and Crawford, 2016). In the near future, unlike what was suggested by Steadman, it is far more likely to observe the democratization of technological means and of the nominal access—the European General Data Protection Regulation (GDPR) represents a first crucial step in this direction—and a restriction of actual access due to the increased difficulty in data computing.

The democratization of the nominal access will have to deal with the rising concerns about privacy. The awareness of great risks for privacy emerged shortly after the diffusion of big data (Bollier, 2010; McDermott, 2017), but with the “collapse of the control zone” (Lagoze, 2014, p. 6) and the normalization of *dataveilance* (van Dijck, 2014), it seemed that big data were destined to bypass all privacy issues anyway: “Google knows what you're looking for. Facebook knows what you like. Sharing is the norm, and secrecy is out” (Preston, 2014).

Nevertheless, this impression faced numerous examples of ethical ambiguity in big data research. Tsvetkov's artistic project *Your Face Is Big Data* showed that anyone can use pictures of random strangers to easily identify their profiles on social networks (Chulkovskaya, 2016). In 2006, a research group from Harvard gathered data about the Facebook profiles of 1,700 unaware students to investigate changes in interests and relationships over time. While the results were published respecting the anonymity of these users (Lewis et al., 2008), it was soon proved that de-anonymization of the employed and publicly available dataset was still possible (Zimmer, 2008; Boyd and Crawford, 2012). In 2016, a study employing geographical data argued that using big data it was possible to give a name and a surname to the anonymous artist known as Banksy (Hauge et al., 2016; Metcalf and Crawford, 2016). After a legal battle that delayed the publication of the article, the authors finally managed to publish and added a short ethical note:

“*the authors are aware of, and respectful of Mr. Gunningham and his relatives and have thus only used data in the public domain. We have deliberately omitted precise addresses”* (Hauge et al., 2016, p. 5).

In the article, graffiti were defined as “terrorism-related acts” and Robin Gunningham was publicly associated with vandalism. Whether Gunningham really is Banksy or not remains unclear. The study was strongly criticized at an ethical level and its methodological validity was questioned. Banksy was obviously not pleased by the article and newspapers started to pester Gunningham and his family, revealing even more about their personal lives and whereabouts. Three years later, Banksy still remains an anonymous artist.

These brief examples clearly show how easily scientific research can harm studied subjects in the Petabyte Age. It is no longer possible to assume that public data are *a-problematic* from an ethical point of view. On the contrary, the availability of data is today a sensitive topic in itself. As for the anonymity and informed consent, things are arguably even more complicated. Small adaptive changes to information privacy law will not suffice since big data offered a radically new perspective on the issue at hand.

**Figure 1:**
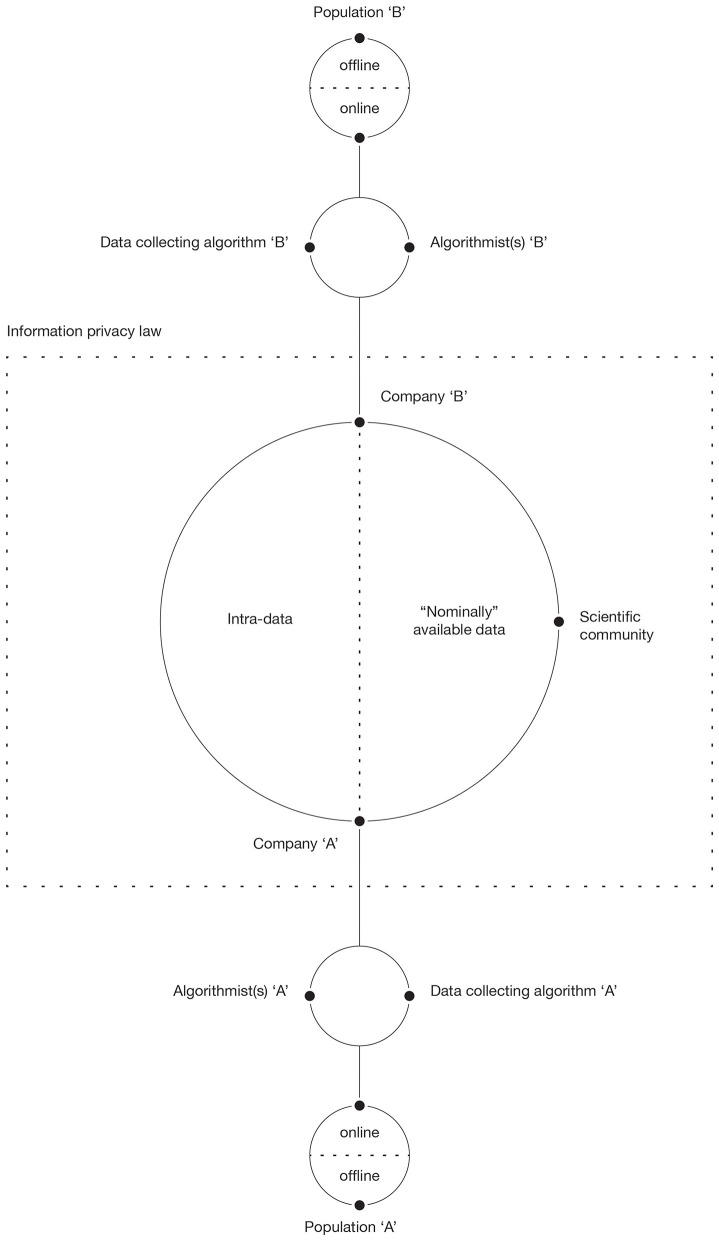
Power dynamics, data collection, and nominal access in big data production.

The main and arguably the more radical effect of big data thus rests at the crossroads between business methods, academic research, emerging laws, and accessibility. Big data entirely changed the rules of the game by redefining power dynamics involved in the processes of data production and knowledge discovery. We therefore propose a theoretical macro-level model (see Figure 1) to orientate future research. The model focuses on collective actors involved in the above-mentioned processes and on the relation they establish between each other.

The center of Figure 1 is occupied by information privacy law that directly affects not only the individual and the collective actors involved in big data usage but also their relations by regulating the access to collected data. The GDPR, for example, dictate that, to collect data of a given kind, there must be a specific business purpose. This poses limitations on the type of information that a company can collect and further accentuate the signal problem discussed above. The renewed attention for privacy and stricter regulations accentuate the compliance to the existing set of rules that pose information privacy law at the center of the complex network of relations in data production (Gruschka et al., 2018). Furthermore, GDPR poses part of the power directly in the consumer's hand who can forbid certain uses of data that he or she is willingly sharing (Yeh, 2018). Moreover, companies involved in data collection may impose further limitations to the nominal access in accordance with current regulations to increase their competitive advantage (Fuchs, 2011). As a result, usually only a part of collected data becomes nominally available. Data unavailable to “outsiders” are here addressed as intra-data. Information privacy laws and business secrecy-related dynamics thus pose a limit to nominal access.

Social scientists are typically not involved in the collection and the storage of big data, which means that they have no control of any kind over the population and the data collection process and experience issues of actual access (Burrows and Savage, 2014; Bonenfant and Meurs, 2020). The entity of this limitation varies across disciplines and does not affect all members of the scientific community equally (Savage and Burrows, 2007; Kelling et al., 2009; O'Leary, 2013). What will be collected, how it will be collected, and how it will be stored and made accessible are thus usually defined within a business context in the interaction between algorithmists and the company that employs them. Secondary data and therefore the analysis of data produced for different purposes is a common thing in research. So, why is it a problem when dealing with big data? Using secondary data, an important part of the researcher's work that typically precedes the analysis is the evaluation of the dataset, aimed at assessing the quality and the appropriateness of data. With big data, the algorithmic opacity and the private nature of relevant information (Burrell, 2016) both negatively affect the actual access, making critical examination of data for scientific purposes significantly more difficult if not nearly impossible (Bonenfant and Meurs, 2020). In this sense, researchers are marginalized and deprived of power, losing control over data, meant as a primary means of knowledge discovery.

Once data collecting algorithms are defined and set in motion, the data collection begins. The target population is distinguished in its online and offline form. While data collecting algorithms capture most of the online information (all *if* we consider only data that algorithms were designed to collect and ignore the issues raised by GDPR), the access to offline data is limited and rather indirect. Since there is no necessary correspondence between online and offline behavior, the collected data tells us more about the online world than about its offline counterpart (see Crawford, 2013; Lewis, 2015; Gransche, 2016). Algorithms improve over time due to the machine learning process and feed “data back to users, enabling them to orient themselves in the world” shaping human agency directly (Kennedy et al., 2015, p. 1; see also Graham, 2018).

While methodological changes produced by big data do not seem to suffice to invoke a whole new paradigm in knowledge discovery (see also Leonelli, 2014), the rise of big data drastically shaped the involved actors and their relations. The scientific community was thrown to the borders of the process, losing the control that it is traditionally used to. In this sense, the fractures between business and science on one side and between business methods and research ethics on the other, joined with issues of nominal and actual access, are causing tensions at an epistemological level and pushing science outside of academia.

## Conclusions

This paper offered an extensive literature review while addressing the problem of defining big data, the harmful diffusion of an objectivistic rhetoric, and the impact of big data on knowledge discovery within the scientific domain. As discussed, many authors repeatedly failed in their attempt to provide big data with a distinctive and unitary status by focusing on the inherent characteristics of big data.

Big and small data continue to be affected by subjective decisions and errors at multiple levels. The intrinsic logical fallacies of the presumed neutrality and exhaustivity in data collection, analysis, and interpretation have been explored and illustrated.

Following Lagoze's (2014) distinction between evolutionary and revolutionary dimensions, big data have been interpreted as a methodological revolution carried over by epistemological and technological evolution. In this sense, we argue, big data are not calling for a radical change of paradigm as other authors claimed but rather for an adaptive redefinition and re-discussion of current standards in social sciences. By shifting the attention from the intrinsic characteristics of the object to the relations established between acting subjects and the object at hand, it becomes possible to trace a demarcation line between small and big data. In fact, the area where big data are provoking major changes, differentiating themselves from the so-called small data, is precisely the one of relations involved in data collection, data storage, and data processing. In this sense, big data are pushing the scientific community to the periphery of the new geography of power dynamics in knowledge discovery and entirely redesigning its landscape while changing stakeholders, gatekeepers, and even the rules of the game.

The widespread talk about “revolution” placed big data in a sort of virgin territory where everything was possible. By emphasizing sources of continuity, we tried to bring the debate back to the third paradigm to start anew from a common ground. It is undeniable that the developments observed during the past two decades cannot always and entirely be dismissed as simple evolutionary and adaptive changes, and yet neglecting these aspects in favor of the distracting twinkle of novelty establishes the risk to undermine interdisciplinary cooperation and promotes structural shortsightedness. In the European context, this will arguably be even more important in future years given the recent attempt of the European Commission (2020) to pursue an “ecosystem of trust” through a “coordinated European approach […] on the better use of big data” in artificial intelligence research. Once the hype is over, the scientific community will have to face the fact that the changing power dynamics has led to the privatization of relevant information. The talk about transparency, representativeness, robustness, privacy, replicability, and comparability will thus have to resume, not to satisfy some remote theoretical need disconnected from reality but to establish acceptable practices and standards in a mutated context and to provide an effective tool for policy-making. To do so, at least some degree of agreement about what actually constitutes the subject of the discussion will be needed.

## Author Contributions

The manuscript has been written by DB under the supervision of DR. Both authors contributed to the article and approved the submitted version.

## Conflict of Interest

The authors declare that the research was conducted in the absence of any commercial or financial relationships that could be construed as a potential conflict of interest.

## Footnotes

^1^Alternative sources report the existence of 300 exabytes of data in 2007 and 1,200 exabytes in 2013 with a decrease of non-digital data from 7% to a mere 2% (Mayer-Schönberger and Cukier, 2013a).
